# The application of quality control circle in the management of narcotic drugs and first-category psychotropic drugs

**DOI:** 10.1097/MD.0000000000045543

**Published:** 2025-12-12

**Authors:** Yanli Ma, Xiaobei Ma, Siyi Yan, Hongmei Duan

**Affiliations:** a Beijing Tsinghua Changgung Hospital, School of Clinical Medicine, Tsinghua University, Beijing, China; b Beijing University of Chinese Medicine, Beijing, China.

**Keywords:** management, narcotic drugs and first category psychotropic drugs, quality control circle

## Abstract

This study explores the application effect of “quality control circle” (QCC) activities in the management of narcotic and 1st-category psychotropic drugs (anesthetic drugs). A QCC management group was established based on standardized methods and procedures. The theme of the group was to improve the standardization rate in the management of anesthetic drugs. The group analyzed the current situation of anesthetic drug management in a tertiary public hospital in Beijing, China. The plan-do-check-act cycle was applied to implement improvement measures. The standardization rate, management time, and healthcare satisfaction of anesthetic drug management were compared before and after QCC management. The standardization rate of anesthetic drug management increased from 95.83% to 99.01% after QCC, which showed that staff satisfaction was higher after QCC management than before (*P* < .01). The time anesthesia nurses spent each day managing anesthetic drugs was also significantly reduced, from 72.68 ± 15.41 minutes to 51.74 ± 6.92 minutes (*P* < .01). The application of QCC management not only increased the standardization rate of anesthetic drug management, but it also improved job satisfaction and reduced the time spent managing anesthetic drugs.

## 1. Introduction

Anesthetic drugs play an important role in perioperative treatment and can be used for analgesia, providing comfort to patients, and facilitating postoperative recovery; however, standardizing the management of anesthetic drugs and monitoring their use are key to ensuring safe and effective clinical practice. For historical reasons, China is one of the countries with strict regulations on anesthetic drugs.^[1–3]^ The state has established laws and the health regulator has set up very strict systems and requirements for hospitals.^[4,5]^ However, the risk of abuse of medical anesthetic drugs still exists in China. The proportion of abusers of narcotic drugs and psychotropic substances for medical use in the monitored population amounted to approximately 3.7%.^[6]^ Hospitals face many challenges in the management of anesthetic drugs, including inventory management, medication safety, human resource management, and other areas.

Quality control circle (QCC) is widely used as quality management tools in a variety of fields, and are based on team participation, problem identification, and resolution aimed at improving quality and performance within an organization.^[7–9]^ Through the application of QCC, anesthesiologists and nurses can participate in the management of anesthetic drugs, thus improving the overall management effect.

The purpose of this paper is to explore the application of QCC in the management of anesthetic drugs in Chinese hospital anesthesiology departments and to assess its impact on quality control and patient safety. By applying QCC methodology, members can analyze potential problems and risks and explore feasible solutions. Circle members proposed improvement measures such as e-books and information management.^[10–13]^ Due to the complexity of the management of anesthetic drugs, QCC also promotes knowledge sharing, collaboration, and cross-functional cooperation between hospital departments, improving efficiency and patient satisfaction. This study aims to provide useful insights for relevant practitioners and to further promote the development and innovation of anesthetic drug management in hospitals.

## 2. Data and methods

### 2.1. General information

From April 2023 to October 2023, the anesthesia department of a tertiary general public hospital in Beijing was selected for this study. There were 40 anesthesiologists and 41 anesthesia nurses in the department, with 1 nurse dedicated to administering anesthetic drugs. All 81 doctors and nurses participated in the study, and no new staff members joined during this period. The same group of people were surveyed before and after the QCC management. All staff were informed of the trial, participated voluntarily, and complied with the tenets of the Declaration of Helsinki.

### 2.2. Formation of QCC

**Figure 1.**
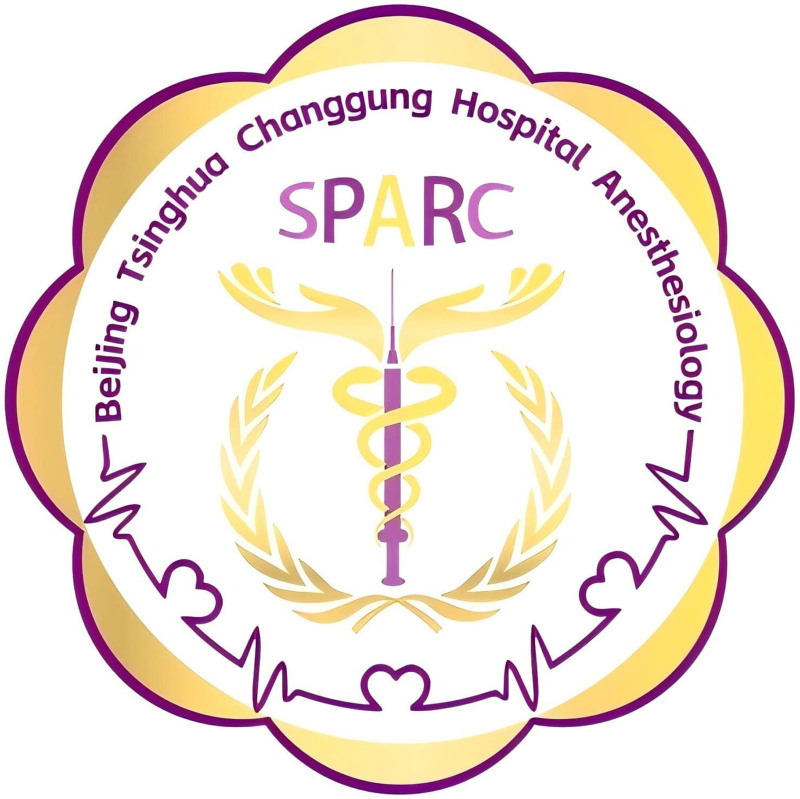
Circle theme: safe, painless, anesthesia, recovery, and care.

The QCC group consisted of 10 members, including 1 anesthesiologist, 6 nurse anesthetists, and 3 other members from the Medical Management Department, the Pharmacy Department, and the Information Department. This QCC activity generally followed the Plan–Do–Check–Act cycle and included 10 basic steps: select theme, plan activity, assess status quo, set goal, analyze, formulate strategy, execute and evaluate, verify results, standardize, and review and improve. The QCC group that was set up was named the “SPARC Circle” (standing for Safe, Painless, Anesthesia, Recovery, and Care) (see Fig. 1).

### 2.3. Methods

The control group was managed with routine measures, while the experimental group was managed with QCC management. The baseline survey was conducted by a nurse responsible for managing anesthetic drugs. After the QCC intervention, data were collected by another anesthesia nurse at the N3 level. Statistical analysis was carried out by a nurse with a postgraduate degree. The subjects, researchers, and data analysts were blinded to the grouping conditions before and after the experiment. Surveys conducted before and after the implementation of the QCC assessed the standardization rate of anesthetic drug management, staff satisfaction, knowledge acquisition rate, and the amount of time anesthesia nurses spent each day on anesthetic drug management.

The study design and protocol were approved by the Beijing Tsinghua Changgung Hospital Ethics Committee (No. 23043-01-10) and was conducted in accordance with the Declaration of Helsinki and other relevant guidelines and regulations. No patients were involved in this study.

#### 2.3.1. Select theme

All members listed problems that needed to be solved in work by the brainstorming method and evaluated 5 items of superior policy, urgency, feasibility, circle ability, and effectiveness. Finally, calculated the highest score by using the 5-3-1 scoring method, which was determined to be the theme of this activity: to improve the standardized rate of the management of anesthetic drugs. The standardization was defined as: borrow and return compliance system; double operation in the process; destruction of residual anesthetic drugs comply with requirements; accounts are in line with the physical; the prescription is complete and correct; forms for using records are standardized and correct; the account books are correct; there is no breakage or loss of anesthetic drugs.

#### 2.3.2. Plan activity

**Figure 2.**
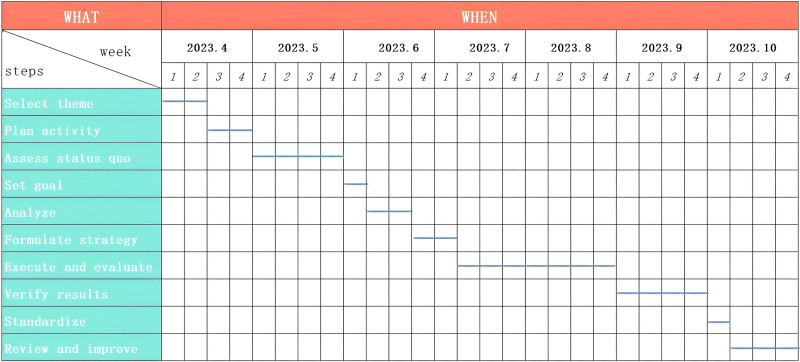
Active Gantt chart. According to the PDCA procedure, the group discussed and formulated a detailed activity schedule, evaluated the implementation steps, selected the person responsible for each step, drew a Gantt chart, and carried out the activities according to the plan. PDCA = Plan–Do–Check–Act.

The group discussed and formulated a detailed activity schedule, evaluated each steps, selected the person responsible, drew a Gantt chart (see Fig. 2), and carried out the activities according to the plan.

According to the Plan–Do–Check–Act procedure, the group discussed and formulated a detailed activity schedule, evaluated the implementation steps, selected the person responsible for each step, drew a Gantt chart, and carried out the activities according to the plan.

#### 2.3.3. Investigation of the status quo and goal setting

To fully investigate the current status of anesthetic drug management, a cross-sectional questionnaire survey was conducted among the medical and nursing staff of the Department of Anesthesiology in April 2023. All questionnaire tools were internally designed. The staff’s satisfaction questionnaire included the following items: satisfaction with the receiving process, satisfaction with the return process, satisfaction with the destroying the residual liquid, and satisfaction with management training. The Cronbach α coefficient of the staff satisfaction questionnaire was 0.786, and the item-level content validity indices ranged from 0.75 to 1.00. The results showed that the average satisfaction level was only 75.7%. Problems over a 4-week period were collected and analyzed using a checklist. All prescriptions for patients who received anesthetic drugs during surgery were included in the analysis. During the investigation period, there was no significant fluctuation in the monthly number of surgical patients or anesthetic drug prescriptions. Among the 10,158 prescriptions reviewed, the average standardization was 95.8%, and the average checking time of anesthetic management nurses was 72.7 minutes per day.

**Figure 3.**
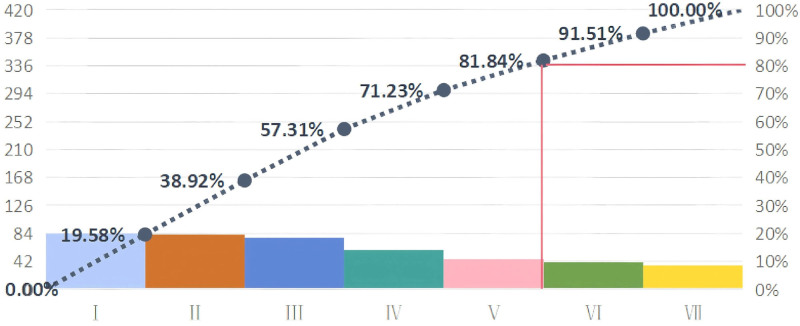
Pareto chart before improvement. (I) Irregular usage record form. (II) Irregular return of anesthetic drugs. (III) Irregular account book registration. (IV) Unqualified prescription. (V) No charge. (VI) Irregular claim of anesthetic drugs. (VII) The accounts do not tally.

According to the data collected, the pre-platonic improvement was made (see Fig. 3), and 81.84% of the cases were irregular usage record form, irregular return of anesthesia drugs, irregular account book registration, unqualified prescription and no charge. These 5 items were prioritized for improvement based on the “Pareto chart 80/20.”

Based on the members’ seniority, education, improvement ability, and QC circle experience value, the capacity of this circle was calculated to be 74.8%. To determine the target value for improvement, the following formula was used: target value = current status value + (standard value − current value) × improvement focus × circle capacity = 95.8% + (100 − 95.8) × 81.84% × 74.8% = 98.37%. Therefore, the goal of improvement was to increase the standardization rate of anesthetic drug management in the anesthesia department from 95.8% to 98.4% by October 2023.

#### 2.3.4. Strategies and implementation

**Figure 4.**
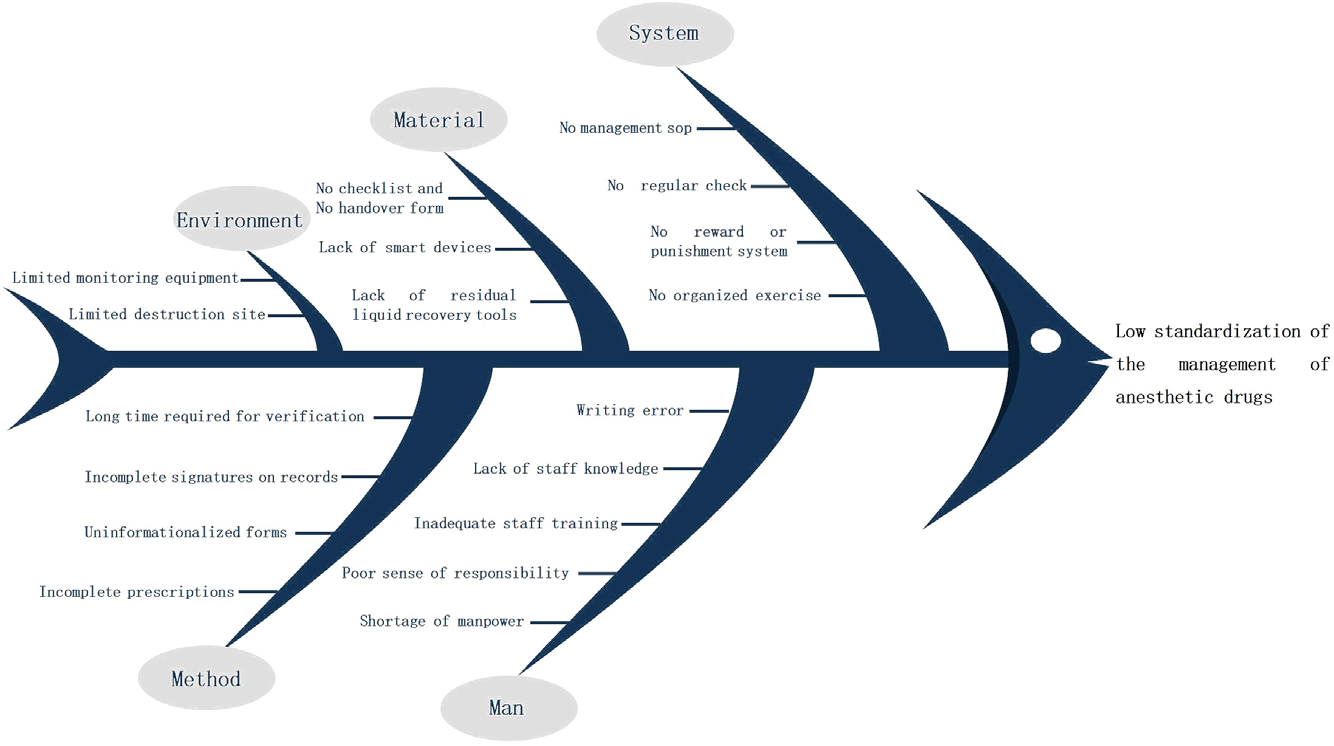
Fishbone diagram illustrating the important contributing factors.

Through a literature review, questionnaires, and brainstorming, we analyzed the causes in terms of personnel, systems, materials, methods, and environment, and drew a fishbone diagram (see Fig. 4).

**Figure 5.**
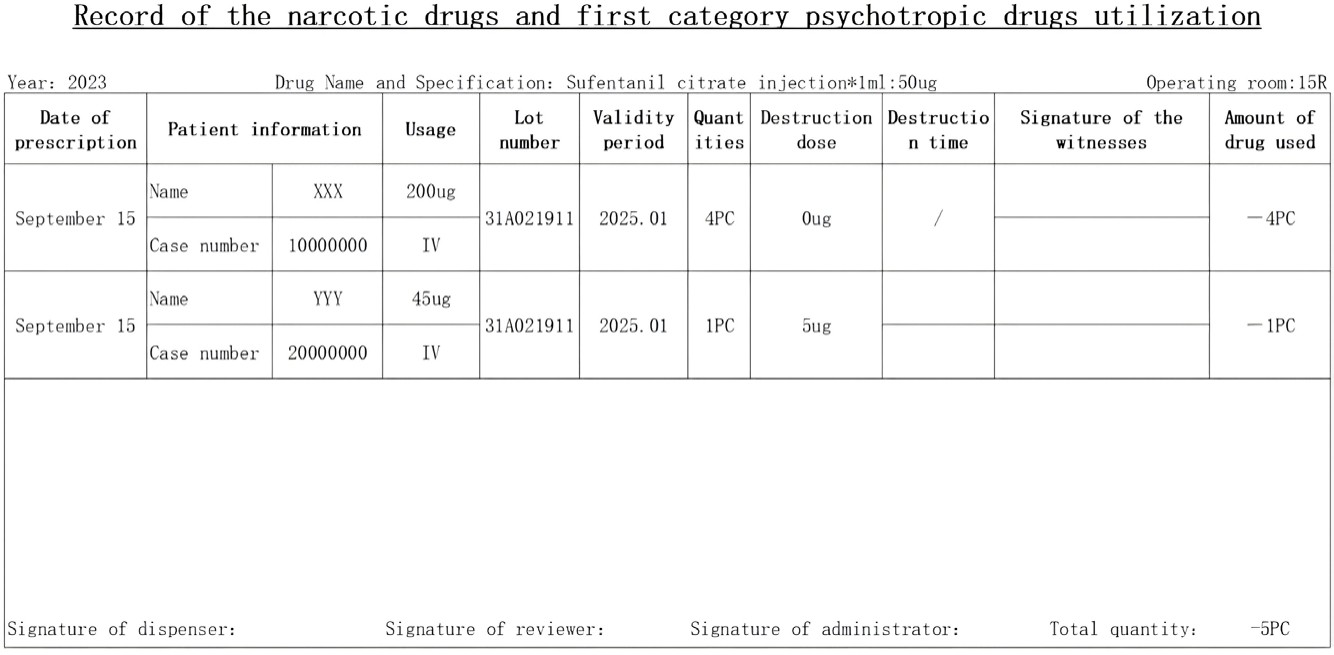
Record of anesthetic drug utilization.

**Figure 6.**
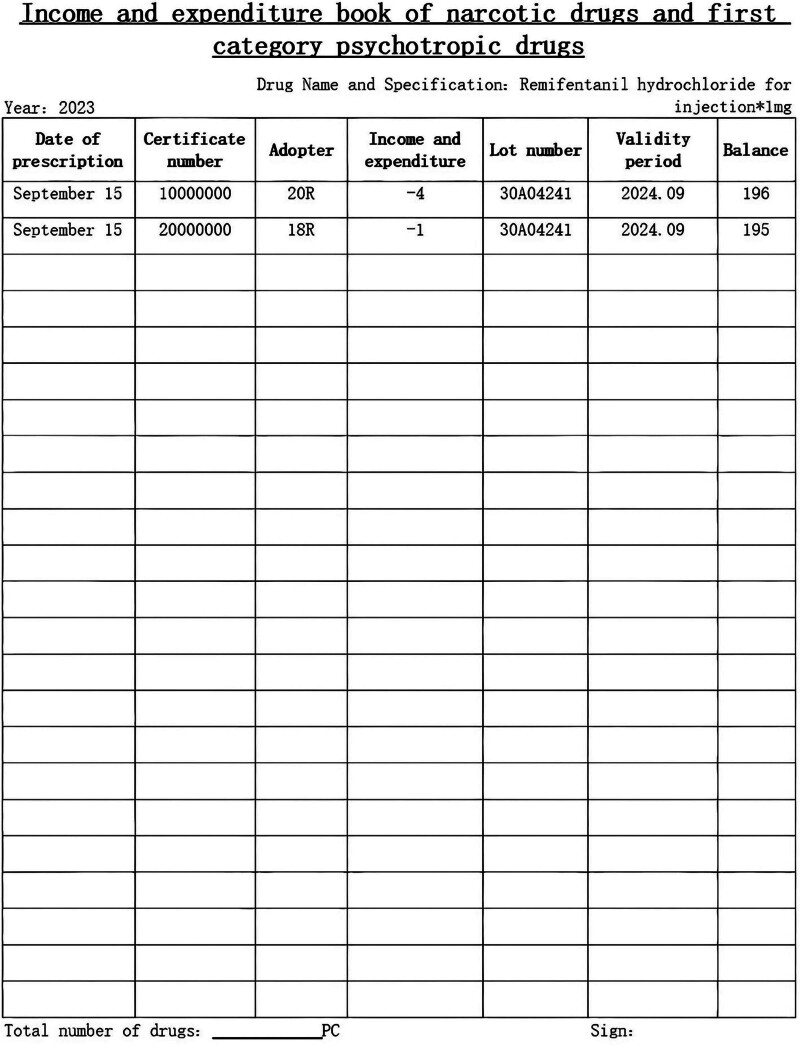
Income and expenditure books.

**Figure 7.**
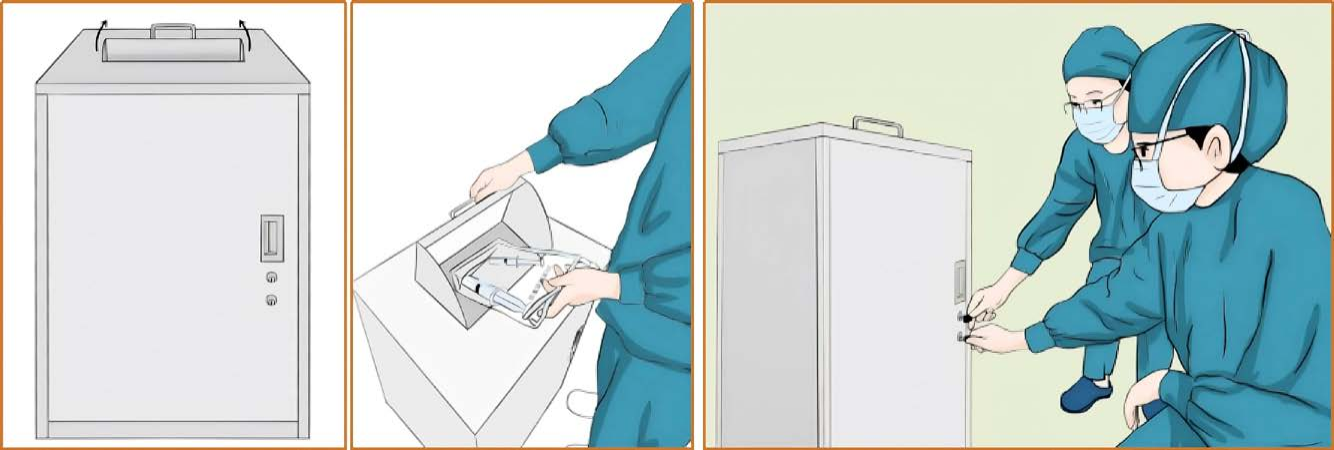
One-way drop-off storage box for the residual liquid.

After brainstorming discussions among the group members, implementable countermeasures were proposed, and 6 countermeasures were formulated for the above problems, all of which were scored and evaluated using a secret ballot. The evaluation items were divided into importance, urgency, and capacity. The specific implementation was as follows: the WeChat app was applied for training on anesthetic drug management. This app can be viewed using one’s phone after providing an electronic signature at any time, or the data can be exported as a PDF file. Drills for emergencies to enhance staff’s ability to deal with unusual situations were carried out; these emergencies included medicine cabinets that could not be locked or opened, broken vials, and the loss of anesthetic drugs. Informatization of prescriptions for anesthetic drugs: automatic capture of patient information, drug name, dosage, route, batch number, expiration date, etc, from within the HIS system or the anesthesia system. The electronic records of anesthetic drugs were used to generate a form with patient information, administered dosages, destruction doses, and quantity of drugs used, which can be printed and verified (see Fig. 5). An income and expenditure book was then generated based on this form to facilitate drug counting and handover (see Fig. 6). To improve the destruction of the residual liquid of anesthetic drugs, members brainstormed and designed a 1-way drop-off storage box for the residual liquid that was inspired by old clothes recycling boxes. This tool facilitates the centralized destruction of residual liquids by 2 people, overcoming the problem of insufficient manpower (see Fig. 7). The utility model patent has been approved (ZL 2023 2 3144989.7). It avoids the loss of residual liquid and facilitates centralized destruction. Specialized people check and count the problems in the management of anesthetic drugs and organize targeted retraining.

## 3. Results

Data were statistically analyzed using SPSS 25.0 (IBM Corp., Armonk). Measurement data were expressed as the mean ± standard deviation (X ± S) and analyzed using Student *t* test. The count data were compared using the *χ*^2^ test. *P* < .01 was considered to indicate statistical significance.

### 3.1. Comparison of the rate of standardization of the management of anesthetic drugs before and after the implementation of the QCC

This study counted 10,158 and 11,610 prescriptions for anesthetic drugs over a 4-week period before and after QCC activities, respectively, and found a significant increase (*P* < .05) in the rate of standardization of the management of anesthetic drugs after the QCC (99.01%) compared to the rate before (95.83%) (see Table 1). The standardization rate continued to be tracked monthly as a nursing quality indicator and has remained above 99% each month.

**Table 1:** Comparison of the rate of standardization before and after the QCC.

	Before/number of prescriptions (n = 10,158)	After/number of prescriptions (n = 11,610)	95% CI	Phi	χ^2^	*P*
Substandard quantity	424 (4.17)	115 (0.99)	−0.036 to −0.028	0.102	227.383	.000*
Standardized quantities	9734 (95.83)	11,495 (99.01)				

QCC = quality control circle.

* *P* < .01.

### 3.2. Comparison of the staff’s satisfaction before and after the implementation of the QCC

Comparing the satisfaction with the management of anesthetic drugs before and after the QCC activities, it can be seen that the staff’s satisfaction with the process of receiving, returning, and destroying the residual liquid increased significantly (75.56%/88.89%, 72.84%/91.36%, 69.88%/93.33%, all *P* < .05). There was no significant difference in satisfaction with training in the management of anesthetic drugs (84.69% vs 89.38%, *P* = .08) (see Table 2).

**Table 2:** Comparison of the staff’s satisfaction before and after the QCC.

		Before/number of staff (n = 81)	After/number of staff (n = 81)	95% CI	Phi	*χ* ^2^	*P*
Not satisfied		40 (12.35)	0 (0.00)	−0.159 to −0.088	0.256	42.632	.000*
Satisfied		284 (87.65)	324 (100.00)				
Staff’s satisfaction with the process of receiving	Not satisfied	12 (14.81)	0 (0.00)	−0.240 to −0.081	0.283	12.960	.000*
	Satisfied	69 (85.19)	81 (100.00)				
Staff’s satisfaction with the process of returning	Not satisfied	14 (17.28)	0 (0.00)	−0.255 to −0.090	0.308	15.324	.000*
	Satisfied	67 (82.72)	81 (100.00)				
Staff’s satisfaction with the process of destroying the residual liquid	Not satisfied	11 (13.58)	0 (0.00)	−0.210 to −0.061	0.270	11.801	.001
	Satisfied	70 (86.42)	81 (100.00)				
Staff’s satisfaction with the training of management	Not satisfied	3 (3.70)	0 (0.00)	−0.078 to 0.004	0.137	3.057	.080
	Satisfied	78 (96.30)	81 (100.00)				

QCC = quality control circle.

* *P* < .01.

### 3.3. Comparison of the rate of knowledge acquisition before and after the implementation of the QCC

The correct rate of knowledge of anesthetic drugs among doctors and nurses was 66.43 ± 7.59% before QCC activities. The correct rate after the activity was 87.23 ± 5.41%. A comparison of the correct rate (*P* < .05) was considered statistically significant (see Table 3).

**Table 3:** Comparison of the rate of knowledge acquisition before and after the QCC.

	Before/number of staff (n = 81)	After/number of staff (n = 81)	95% CI	Cohen’s d	*t*	*P*
Examination scores	66.43 ± 7.59	87.23 ± 5.41	−22.849 to −18.756	3.157	−20.089	.000*

QCC = quality control circle.

* *P* < .01.

### 3.4. Comparison of the time consumed before and after the implementation of the QCC

The daily checklist took 72.68 ± 15.41 minutes before the QCC activity and 51.74 ± 6.92 minutes after the activity. This shorter time was statistically significant (see Table 4).

**Table 4:** Comparison of the time consumed before and after the QCC.

	Before/number of working days counted (n = 21)	After/number of working days counted (n = 23)	95% CI	Cohen’s d	*t*	*P*
The time consumed	72.68 ± 15.41	51.74 ± 6.92	15.331 to 26.557	1.605	7.464	.000*

QCC = quality control circle.

* *P* < .01.

### 3.5. Radar map of invisible results

**Figure 8.**
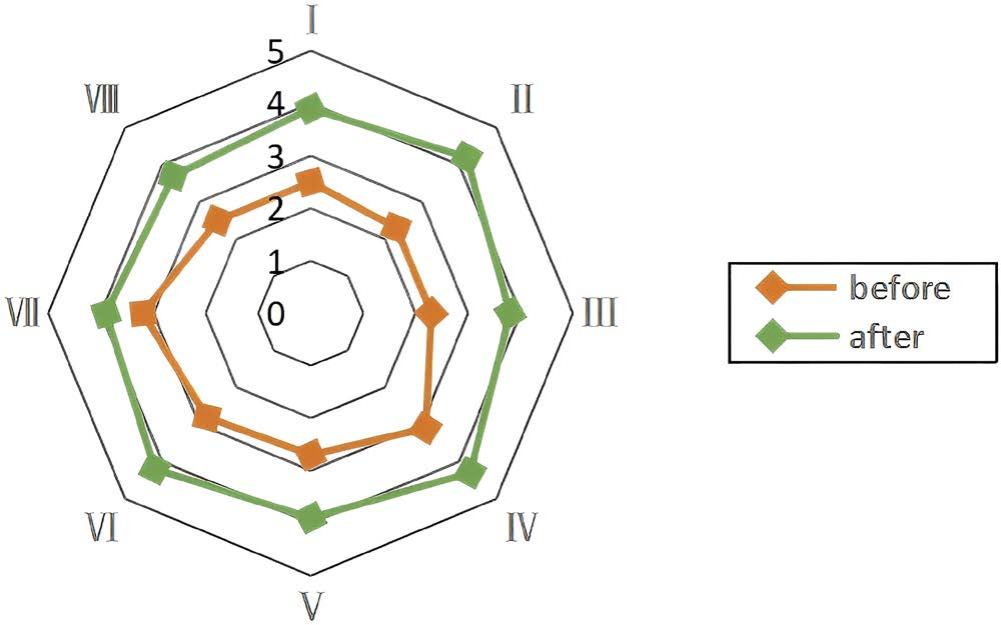
Radar map of invisible results. (I) Spirit of leadership. (II) Quality control technique. (III) Sense of accomplishment. (IV) Sense of honor. (V) Team cohesion. (VI) Enthusiasm. (VII) Sense of responsibility. (VIII) Cooperative ability.

Circle members reported improvements in their spirit of leadership, quality control techniques, sense of accomplishment, sense of honor, cohesion, enthusiasm, sense of responsibility, and cooperation after implementing the QCC project, as shown in Figure 8.

## 4. Discussion

The problem of drug abuse is attracting increasing attention worldwide, not only as a public health hazard, but also as an important factor affecting the safety of society. The abuse of anesthetic drugs has become a global problem that poses a major threat to the survival and development of mankind and has attracted the attention of governments.^[14]^ The Chinese Government has long recognized the importance of anesthetic drug management, and the control of anesthetic drugs has gone through a process of change from lawlessness to lawfulness, from administrative control to legal control. The importance of the management of anesthetic drugs is self-evident. At present, the management modes of different hospitals are not the same, and there is a lack of a perfect, efficient, and standardized management process for parallel promotion.

A QCC can be characterized as a collaborative assembly of individuals operating within the same professional environment and tasked with analogous roles.^[15,16]^ It is a highly applied quality control method that aims to continuously improve work processes and service quality through a high degree of participation and collaboration among staff. The basic principles are as follows: employee participation: a QCC encourage employees to actively participate in the quality improvement process, allowing them to discover management problems and propose solutions. Teamwork: QCC activities require team members to cooperate with their leaders. Periodic meetings: regular meetings were held to discuss the current quality issues and propose suggestions. Data analysis: data analysis is performed to understand the root causes of problems and evaluate the effectiveness of improvement measures. Continuous improvement: the goal of a quality control cycle is to achieve continuous improvement.^[17–20]^ The activity of the QCC has been gradually extended to the field of hospital management in recent years, and participants can learn about scientific quality management and improve their work efficiency. QCC can also improve the quality of medical services.^[21]^

In order to solve the problems of time-consuming and laborious checking of the management of anesthetic drugs, low staff satisfaction, incorrect prescription and utilization records, more errors and low efficiency of manual registration, and cumbersome and error-prone processes, QCC was chosen as the process management and problem-solving technique in this study. The results showed that the standardized rate of administration of anesthetic drugs was increased through QCC activity.

Moreover, the QCC activity in the department led to improvements in the comprehensive ability of the QCC team, such as in the use of QCC techniques, teamwork, professional knowledge, and communication and coordination ability. The results suggest that QCC activities not only bring about improvement in the quality of clinical management, but also explore the potential of employees, fully mobilize their work motivation, and improve their sense of teamwork, which is worth promoting in the medical field.

## 5. Conclusion

In summary, the QCC group improved the standardization and efficiency of anesthetic drug management in the Department of Anesthesiology by organized training, the digitization of drug prescriptions, the use of records and books, the design of tools for residual liquid destruction, and other improvement measures. This study shows that the use of QCC activities to standardize the management of anesthetic drugs can effectively improve the comprehensive management level and guarantee the safety of clinical drug use. Therefore, the results of the present study can be widely applied.

At present, there is no fixed model to follow for the management of anesthetic drugs in medical institutions. Future improvement should focus on summarizing standard processes through practical experience and increasing investments in information technology and intelligent equipment. These steps will help ensure the rational application of anesthetic drugs in clinical practice and simultaneously eliminate the hidden dangers of abuse.

## Acknowledgments

We thank LetPub (www.letpub.com.cn) for its linguistic assistance during the preparation of this manuscript.

## Author contributions

**Conceptualization:** Yanli Ma, Xiaobei Ma, Siyi Yan.

**Formal analysis:** Yanli Ma.

**Supervision:** Hongmei Duan.

**Writing – original draft:** Yanli Ma.

**Writing – review & editing:** Xiaobei Ma, Siyi Yan.
